# Establishment of Pluripotent Cell Cultures to Explore Allelopathic Activity of Coffee Cells by Protoplast Co-Culture Bioassay Method

**DOI:** 10.3390/plants9091170

**Published:** 2020-09-09

**Authors:** Shinjiro Ogita, Muchamad Imam Asrori, Hamako Sasamoto

**Affiliations:** 1 Department of Local Resources, Faculty of Bioresource Sciences, Prefectural University of Hiroshima, Shobara 727-0023, Japan; 2 Program in Biological System Science, Graduate School of Comprehensive Scientific Research, Prefectural University of Hiroshima, Shobara 727-0023, Japan; rori.muchamad17@gmail.com; 3 Faculty of Agriculture, Tokyo University of Agriculture and Technology, Fuchu 183-8509, Japan; sasamoto@ynu.ac.jp

**Keywords:** allelopathic activity, coffee, direct somatic embryogenesis, pluripotency, protoplast co-culture

## Abstract

We focused on the demonstration of a new pluripotent coffee cell culture system to control the growth and metabolic functions. Somatic cells in the epidermal layer of in vitro somatic embryos (SEs) of *Coffea canephora* expressed higher pluripotency to produce secondary SEs than primary or secondary meristematic tissue. SEs were ideal explants to selectively induce functionally-differentiated cell lines, both non-embryogenic callus (nEC) and embryogenic callus (EC). The protoplast co-culture bioassay method was used to explore allelopathic activity of these cultured coffee cells. Cell wall formation of lettuce protoplasts varied after five days of co-culture. A strong stimulative reaction was observed at lower nEC protoplast densities, whereas growth was inhibited at higher densities. The reaction of lettuce protoplasts after 12 days of co-culture was recognized as an inhibitory reaction of colony formation.

## 1. Introduction

Generally, two primary meristems, the shoot apical meristem (SAM) and root apical meristem (RAM), located at the tip of the stem and root, respectively, are responsible for plant longitudinal growth [1]. Additionally, plants develop a secondary stem, called the cambium, that allows them to grow radially and contributes to the vasculature and mechanical support structures [2,3]. One of the fundamental issues in plant science is to unveil the regulatory mechanisms of the series of functional changes that occur in plant meristems during their development [4].

The classical concept for this issue is “totipotency”, which is the inherent capacity of a plant cell to develop into a whole plant. Numerous studies have been carried out to characterize the totipotency of plant cells in terms of morphology, physiology, and molecular biology [5]. In this context, in vitro somatic embryogenesis may be a practical model for the expression of totipotency based on the capacity of an embryogenic cell to regenerate and develop into a somatic embryo that can show the functional changes of the SAM, RAM, and cambium under certain conditions [6].

The evolving concept of stem cells is also essential to elucidate the process of specialization in a plant cell, especially in cell and tissue cultures. Stem cells proliferate in an undifferentiated state and can develop into any type of differentiated cell or tissue. In glossaries, for example [7], stem cell developmental potency is classified into several types: unipotent, a stem cell able to develop into only one cell or tissue type; multipotent, a stem cell able to develop into more than one cell type of the plant body; pluripotent, a stem cell able to develop into most, but not all, cell types that constitute the body; and totipotent, a stem cell able to develop into all cell types. These are reflected by case studies that use various cell and tissue culture methodologies, such as callus culture, tracheary element formation, adventitious bud/root formation, and somatic embryogenesis [8,9,10,11]. However, to date, there are no definite and highly efficient standards for the regulation of stem cell development in in vitro plant cell and tissue cultures. Therefore, we focused on the demonstration of a new pluripotent cell culture system to control the growth and metabolic functions of the target plant. Coffee (*Coffea canephora*) was selected for the present study. It is one of the most important commodities worldwide, and has a great economic impact in many countries [12]. One of the major secondary metabolites of coffee, caffeine (1,3,7-trimethylxanthine), is an allelochemical that affects plant distribution, community formation, intercrop evolution, and biodiversity conservation, which are topics of international interest [13].

As efficient methods for indirect and direct somatic embryogenesis in coffee have already been established [14], we describe here the selective induction of two pluripotent cultured coffee cells, non-embryogenic (nEC) and embryogenic (EC) calli, from somatic embryos and examination of the relationship between morphological changes and metabolic functions. Furthermore, as a new in vitro bioassay for allelopathy, the protoplast co-culture method, has been developed [15] to contribute to the study of allelochemicals and mechanisms of allelopathy, we applied this unique methodology to evaluate the selectively-induced cultured coffee cells using our new pluripotent cell culture system.

## 2. Results

### 2.1. Establishment of Pluripotent Coffee Cell Cultures

**Figure 1:**
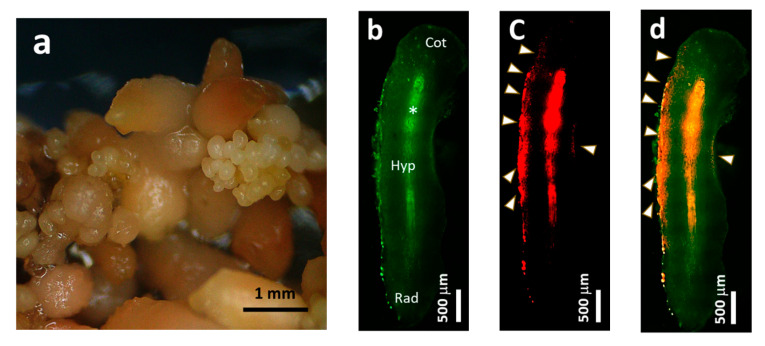
Direct somatic embryogenesis of *Coffea canephora*. (**a**) Macroscopic image of somatic embryos (SEs). (**b**) Autofluorescent image of a somatic embryo (SE) under B-excitation light. Cot = cotyledon, Hyp = hypocotyl, Rad = radicle, Asterisk (*) = vascular bundle region. (**c**) The image of the propidium iodide (PI) staining SE under G-excitation light. (**d**) The merged image of (**b**,**c**). Arrowheads in (**c**,**d**) indicate mitotically activated region.

Direct somatic embryogenesis was induced in coffee leaves, and maintained in the same medium (Figure 1a). The histological characteristics of the epidermal cell layer of coffee somatic embryo (SE) were recognized by propidium iodide (PI) staining and fluorescence microscopy. Yellow–green autofluorescence was identified in the cell walls of the SE, and strong red fluorescence was observed along the cells of the epidermal layer (Figure 1b–d), which indicated the existence of specialized cells with high mitotic activity without any callusing. As secondary SEs were directly induced from the surface of matured SEs during maintenance subcultures (Figure 1a), SEs are highly pluripotent and ideal tissues to selectively induce functionally-differentiated cell lines, nEC and EC calli.

**Figure 2:**
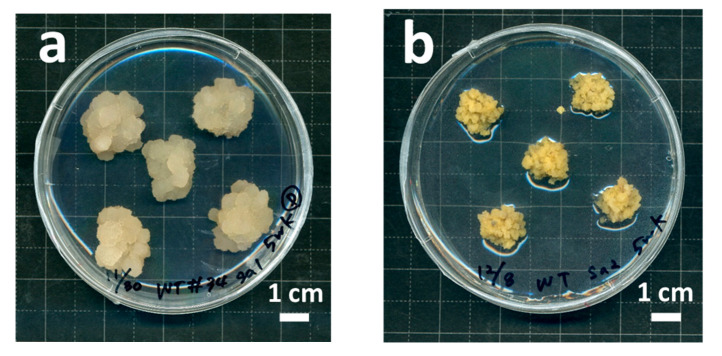
A new pluripotent *C. canephora* cell culture derived from somatic embryos. (**a**) Non-embryogenic callus (nEC). (**b**) Embryogenic callus (EC).

When the coffee SEs were transferred on modified MS medium that contained 10 μM 2,4-D only, nEC, which is soft and white (Figure 2a), actively proliferated, whereas the induction of EC, which is smooth and yellow to yellow–white (Figure 2b), was observed on modified Murashige and Skoog (MS) medium that contained 10 μM 2,4-D and 3 μM kinetin. These two prominent cell lines were selected to investigate their growth features.

**Figure 3:**
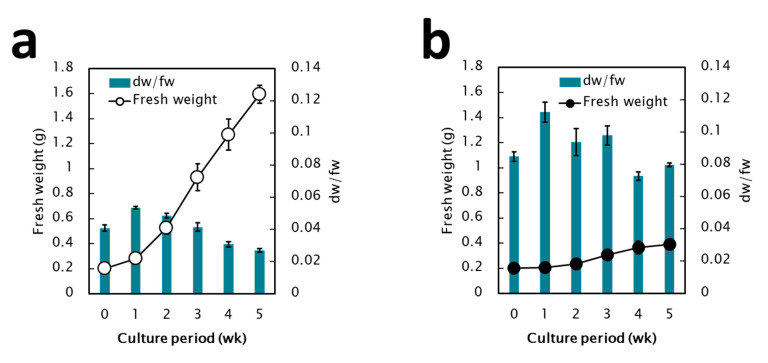
The growth features of non-embryogenic callus (**a**) and embryogenic callus (**b**) during subculture.

**Figure 4:**
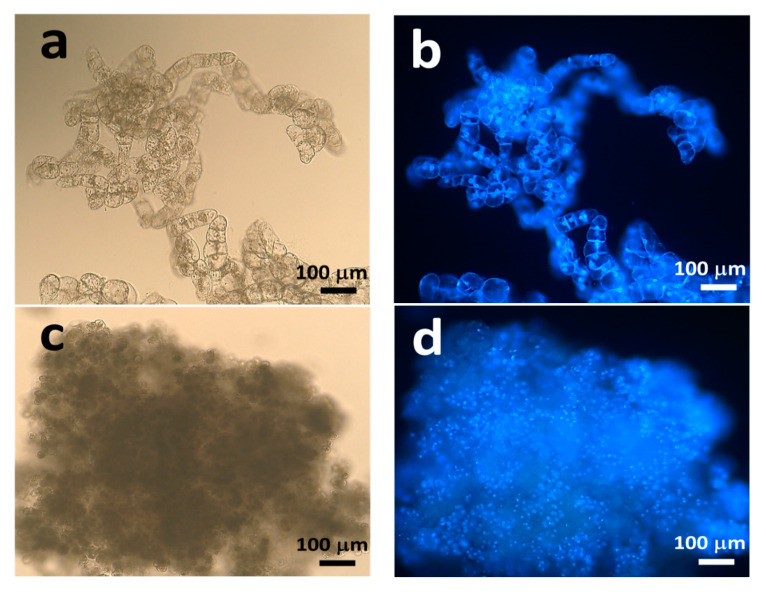
Histological characteristics of non-embryogenic callus (nEC) and embryogenic callus (EC). (**a**) The image of nEC under a bright field. (**b**) The image of nEC stained with 4′-6′-diamidino-2-phenylindole dihydrochloride (DAPI) under U-excitation light. (**c**) The image of EC under a bright field. (**d**) The image of EC stained with DAPI under U-excitation light.

The fresh weight (fw) and the ratio of dry weight (dw) to fw, which represent moisture content, were monitored during 5 weeks of subculture. The nEC showed an 8-fold higher proliferation capacity (Figure 3a), whereas EC showed a twofold higher proliferation capacity (Figure 3b). The ratio of dw/fw in EC was higher than that in nEC (0.08–0.12 and 0.02–0.05, respectively). These differences could be explained by the histological observations with 4′-6′-diamidino-2-phenylindole dihydrochloride (DAPI) staining and fluorescence microscopy (Figure 4a–d). First, the diameter of EC cells (10–15 μm) was less than that of nEC cells (50–80 μm). Second, EC consisted of tiny, dense, cytoplasmic cells with high mitotic activity, whereas nEC consisted of actively proliferating cells with vacuolation.

### 2.2. Protoplast Isolation

**Figure 5:**
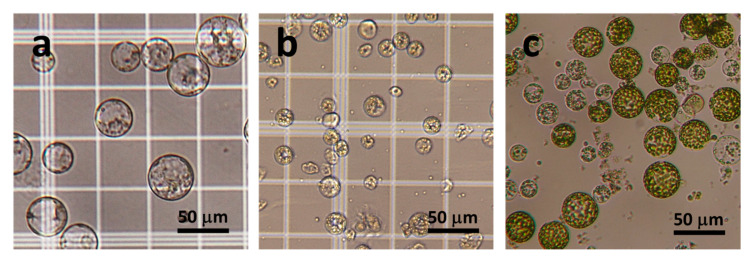
Microscopic characteristics of protoplasts. (**a**) Protoplasts derived from coffee non-embryogenic callus. (**b**) Protoplasts derived from coffee embryogenic callus. (**c**) Protoplast co-culture at 0 day. Green lettuce protoplasts are distinct from whitish coffee protoplasts.

Protoplasts were successfully isolated from coffee EC using the 0.6 M mannitol solution that contained 1% cellulase R10 only. However, the addition of 1% cellulase RS and 1% Driselase 20 was required for the isolation of protoplasts from coffee nEC. By using fluorescein diacetate (FDA) staining, the viability of isolated protoplasts was estimated higher than 90%. The diameter of the EC-derived protoplasts (10–15 μm) was less than that of nEC-derived protoplasts (50–80 μm) (Figure 5a,b). These coffee protoplasts were white–yellow.

### 2.3. Protoplasts Co-Culture

**Figure 6:**
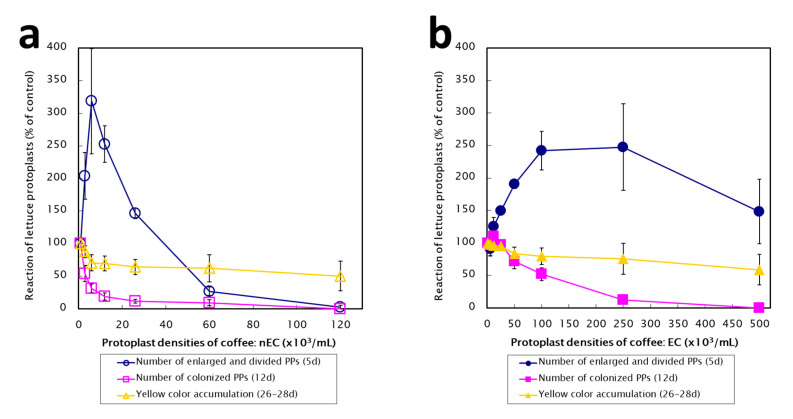
The effects of coffee protoplasts on the growth response of lettuce protoplasts during the co-culture at 5, 12, and 26–28 days. (**a**) Non-embryogenic callus (nEC); (**b**) Embryogenic callus (EC).

Green spherical lettuce protoplasts (stage A) were distinguishable from coffee protoplasts under an inverted microscope (Figure 5c). The effects of coffee protoplasts on the growth response of lettuce protoplasts during the co-culture at 5, 12, and 26–28 days are summarized in Figure 6.

### 2.4. Effect of Coffee Protoplasts on Non-Spherical Enlargement and Cell Wall Formation Stage of Lettuce Protoplasts

Compared to those in the control culture of lettuce protoplasts without the addition of coffee protoplasts, non-spherical enlargement (stage B) and early cell division (stage C) were varied, especially in the case of nEC after 5 days of co-culture. A strong stimulative reaction (150–300% of control; open circle in Figure 6a) occurred at lower protoplast densities (3–26 × 10^3^/mL). In contrast, the growth of lettuce was inhibited at higher densities (60–120 × 10^3^/mL). Almost no growth was observed with 120 × 10^3^/mL nEC. However, in the case of EC, there was no strong inhibition, but a moderate stimulative reaction was observed in almost all conditions (closed circle in Figure 6b).

### 2.5. Effect of Coffee Protoplasts on the Colony Formation Stage of Lettuce Protoplasts

The reaction of lettuce protoplasts after 12 days of co-culture was recorded as the appearance of cell division and colonization patterns (stage D). A strong inhibitory effect (less than 50% of control; open square in Figure 6a) was observed at all tested nEC protoplast densities. In contrast, the reaction of lettuce was gradually inhibited, depending on the EC protoplast density (closed square in Figure 6b).

### 2.6. Effect of Coffee Protoplasts on Yellow Accumulation

The reaction of lettuce protoplasts after 26–28 days of co-culture was measured as yellow color accumulation in lettuce protoplasts, as previously described [15]. The inhibitory effect (50–80% of control; open and closed triangle) was observed after the addition of nEC and EC.

## 3. Discussion

### 3.1. Control of the Pluripotent Cell Culture System through Coffee Somatic Embryogenesis

Somatic embryogenesis was first demonstrated almost 60 years ago in carrots [16]. Based on recent research, it is now known that somatic embryogenesis takes two forms, indirect and direct, referring to the presence or absence of a phase of callus development [14,17,18]. As this technique is mainly used for superior and transgenic plant production, its culture environment is optimized for healthy plant development. In a survey for optimum hormone conditions, unsuitable conditions that degrade embryogenic potency might be rejected. However, we gained insight into the control of development and dedifferentiation in coffee. Generally, plant epidermal cells have multipotency to develop into functionally-specialized cells, such as stomatal guard cells and trichomes [19]. Furthermore, somatic cells in the epidermal layer of in vitro SEs express higher pluripotency to produce SEs than primary or secondary meristematic tissue. SEs are ideal explants to selectively induce functionally-differentiated cell lines, nEC and EC calli, on MS medium in the absence or presence of kinetin. This indicates that the presence of cytokinin is essential to control the pluripotency of SEs.

### 3.2. Allelopathic Activity of Coffee Cells by the Protoplast Co-Culture Bioassay Method with Digital Image Analysis

Two prominent coffee cell lines derived from the same origin showed distinct features in terms of color, size, morphology, and proliferation capacity. When we isolated protoplasts from coffee EC, the required enzyme conditions differed from that for nEC. The cell wall of EC was digested by the addition of 1% cellulase R10 alone. This result indicates that the EC consists of meristematic cells with thin primary cell walls. However, the addition of 1% cellulase RS and 1% Driselase 20 was required for degradation of the cell wall of nEC, which indicates that nEC is already functionally developed, and has a distinct metabolism to EC.

In a previous paper [20], the effects of four synthetic purine alkaloids, caffeine (1,3,7-trimethylxanthine), theobromine (3,7-dimethylxanthine), theophylline (1,3-dimethylxanthine), and paraxanthine (1,7-dimethylxanthine), on the proliferation of lettuce protoplasts were investigated. These chemicals did not show potent stimulatory activity toward lettuce cell enlargement and division, but showed inhibitory activity. The order of inhibition was caffeine > theophylline > paraxanthine > theobromine. In the current study, the frequency of reactions in lettuce protoplasts varied with the culture period and depended on the protoplast density. Interestingly, a strong stimulative reaction (150–300% of control) was recognized as non-spherical enlargement and early cell division at lower nEC protoplast densities (3–26 × 10^3^/mL) after 5 days of co-culture. In the case of EC, a moderate stimulative reaction was also identified in almost all conditions. We consider endogenous amino acids as possible stimulators in nEC and EC for the following reasons. First, this stimulatory activity was not stable during the co-culture, but was degenerative and anabolic to an inhibitor after 12 days of co-culture. As caffeine, a secondary metabolite of coffee and allelochemical, is a derivative of purine nucleotides, amino acids such as glutamine, glycine, and aspartic acid are essential to form the N atoms of the purine ring of caffeine [21]. Second, the nitrogen source (both inorganic and organic) is a key regulator of the in vitro embryogenic response of *Coffea arabica*, as the response varies with the concentrations of nitrogen sources and the nitrate/ammonium molar ratio [22]. Thus, in this study, endogenous amino acid levels might have affected the biological and physiological features of co-cultured cells. In our previous report [23], the level of endogenous glutamine in nEC was ten times higher than that in EC of *Cryptomeria japonica*. The EC of *C. japonica* could maintain only when it was co-cultured with nEC. Third, glutamine is one of the major endogenous free amino acids in calli and protoplasts of plants that show allelopathic activity, such as *Caesalpinia crista*, *Mucuna gigantea*, *Mucuna pruriens*, and *Leucaena leucocephala* [24,25]. In this study, recipient lettuce protoplasts might have used free amino acids from donor coffee protoplasts for their growth in the early stage.

We must focus on the existence of other candidate metabolites in coffee cells. In a previous study, we investigated the allelopathic activity of coffee nEC using the sandwich method [26]. The oven-dried 10 mg callus sample showed a strong inhibition, of more than 85% compared with the control, in the reduction of the elongation of lettuce hypocotyl and root. To clarify the endogenous caffeine level, we analyzed the water extract of *C. canephora* nEC via a reverse-phase HPLC. The endogenous caffeine level averaged 20–60 nmol/100 mg fw. As the moisture content of coffee nEC is 95%, the caffeine content in 10 mg of oven-dried nEC is 40–120 nmol. The effect of this concentration is similar to the effect of synthetic caffeine, with an inhibitory effect on the growth of lettuce protoplasts at 250–1000 μM [20]. Thus, the endogenous caffeine at the level identified in coffee nEC might function as an allelochemical. Chou and Waller [27] have suggested that the phytotoxins present in coffee tissue are caffeine, theobromine, theophylline, paraxanthine, scopoletin, caffeic acid, chlorogenic acid, vanillin, ferulic acid, p-coumaric acid, and p-hydroxybenzoic acid. As our preliminary HPLC measurements also indicated that several unknown metabolites are detectable at 270 nm in wavelength [28], further metabolic profiling [29,30] of coffee cultured cells is demanded for improving our understanding of the function of unknown metabolites.

## 4. Materials and Methods

### 4.1. Establishment of Pluripotent Coffee Cell Cultures

Direct somatic embryogenesis of Robusta coffee (*Coffea canephora*) was established according to Ogita et al. [14]. Briefly, modified half-strength Murashige and Skoog (MS) medium, i.e., half concentrations of inorganic elements and the same concentrations of other elements compared with the original MS medium [31], which contained 20 μM of 2-isopentenyladenine, was used as the induction medium. The sterilized coffee leaves were cut into 10–15 mm^2^ pieces and placed on the induction medium, which was incubated at 25 °C in the dark. Somatic embryos (SEs) were directly formed without producing calli on the cut edges of leaves. SEs were maintained as secondary SEs without callusing for 5 weeks (Figure 1). We selectively induced two prominent cell lines, nEC and embryogenic calli, from this secondary SE culture using MS medium that contained 680 mg L^−1^ KH_2_PO_4_ and 10 μM 2,4-dichlorophenoxyacetic acid (2,4-D) alone, or in combination with 3 μM 6-furfurylaminopurine (kinetin). Two coffee cultured cell lines used in this study were induced by Dr. Shinjiro Ogita. These cell lines are available at the Plant Cell Manipulation Laboratory (https://www.pu-hiroshima.ac.jp/p/ogita/) by making an MTA contract.

### 4.2. Fluorescence Microscopy

The induced secondary SEs were longitudinally cut in half, stained with propidium iodide (PI), mounted on a glass-bottom dish (D11130H, Matsunami, Osaka, Japan) in phosphate-buffered saline, and observed using the z-stack mode of a fluorescent microscope (BZ-X800, Keyence, Osaka, Japan) to characterize the mitotic activity of the epidermal cell layer (epidermis and cortex region). Cultured coffee cells, nEC and EC, were stained with a 2 × 10^−4^% (*w*/*v*) solution of 4′-6′-diamidino-2-phenylindole dihydrochloride (DAPI), and observed, using an inverted fluorescent cell culture microscope (CKX53, Olympus, Tokyo, Japan), to characterize the histological features of these two cell lines.

### 4.3. Protoplast Isolation

Coffee protoplasts were isolated from the nEC cell line by an enzyme combination of 1% cellulase RS and 1% Driselase 20 in 0.6 M mannitol solution during overnight incubation with moderate shaking at 80 rpm using a rotary shaker. The resulting protoplast solution was passed through a 42–80 μm sized nylon mesh, depending on the diameters of protoplasts, and washed with mannitol solution three times by centrifugation at 100× *g* for 5 min. Another coffee protoplast was isolated from the EC cell line by the same protocol with slight modification; the enzyme condition was 1% cellulase R10 without shaking.

Lettuce (*Lactuca sativa* L., cv. Great Lakes 366, Atariya Noen Co. LTD, Chiba, Japan) cotyledon protoplasts were isolated as described previously [15]. Briefly, they were treated with 1% cellulase RS and 1% Macerozyme R10 in 0.6 M mannitol solution. The viability of protoplasts was determined using a fluorescein diacetate (FDA) solution.

### 4.4. Protoplast Co-Culture of Coffee with Lettuce

The protoplast co-culture was performed as described previously [15]. Briefly, the number of protoplasts was counted using a hemocytometer. Final protoplast densities were adjusted as 3 × 10^3^/mL to 1.2 × 10^5^/mL for coffee nEC, 6 × 10^3^/mL to 5 × 10^5^/mL for coffee EC, and 6 × 10^3^/mL to 10^5^/mL for lettuce. Five microliters of each protoplast in 0.6 M mannitol solution were added to 50 μL of liquid MS medium that contained 1 μM 2,4-D, 0.1 μM benzyladenine, 3% sucrose, and 0.6 M mannitol, in a well of a 96-well culture plate (3075, Falcon^®^, Corning, NY, USA). They were cultured at 28 °C in a humid incubator.

The bioassayed data were collected at three different culture periods. Morphological changes in lettuce protoplasts were categorized according to the shapes of cells at four typical growth stages (A–D), as determined by Sasamoto et al. [20]. First, the number of non-spherically enlarged lettuce protoplasts (stage B) and small numbers of divided cells (stage C) that developed from the initial spherical protoplasts (stage A) were counted under an inverted microscope (CK40, Olympus, Tokyo, Japan) after 5 days of culture. Second, the number of divided cells, including colonies composed of four or more cells (stage D), were counted after 12 days of culture. The percentages of the control, without the addition of coffee protoplasts, were calculated and averaged with standard error at lettuce protoplast densities of 6, 12, and 25 × 10^3^/mL. Third, digital imaging analysis of yellow color accumulation in lettuce protoplasts after 26–28 days of protoplast co-culture at different cell densities (12, 25, 50, and 100 × 10^3^/mL) was performed as described previously [15].

### 4.5. HPLC Analysis

*C. canephora* nEC (100 mg fresh weight) was extracted with 0.5 mL distilled water in a microcentrifuge tube, and the supernatant was subjected to a reverse-phase HPLC (column: Cosmosil Packed Column 5C18-MS-II, 4.6 ID × 150 mm (Nacalai Tesque, Inc., Kyoto, Japan); column oven: 40 °C; solvent: 20% methanol; flow rate: 0.6 mL/min; detection: 270 nm) [26].

## 5. Conclusions

This is the first report that suggests a new pluripotent cell culture system to selectively induce non-embryogenic callus (EC) and embryogenic callus (EC) from a somatic embryo of *C. canephora*. These two prominent cell lines are highly applicable to explore the allelopathic activity of coffee cells by the protoplast co-culture bioassay method. This pluripotent cell culture system will provide a valuable framework for conducting a detailed quantitative analysis to identify authentic allelochemicals and unveil the regulatory mechanisms of stimulative and inhibitory activities during cell cultures.
